# Regulation of Retinal Proteome by Topical Antiglaucomatous Eye Drops in an Inherited Glaucoma Rat Model

**DOI:** 10.1371/journal.pone.0033593

**Published:** 2012-07-05

**Authors:** Maurice Schallenberg, Verena Prokosch, Solon Thanos

**Affiliations:** 1 Institute of Experimental Ophthalmology, School of Medicine, University Clinics Münster and Interdisciplinary Centre for Clinical Research (IZKF), Münster, Germany; 2 Department of Ophthalmology, University of Duisburg-Essen, University Hospital Essen, Essen, Germany; University of Oldenburg, Germany

## Abstract

Examination of the response of the retinal proteome to elevated intraocular pressure (IOP) and to the pharmacological normalization of IOP is crucial, in order to develop drugs with neuroptorective potential. We used a hereditary rat model of ocular hypertension to lower IOP with travaprost and dorzolamide applied topically on the eye surface, and examine changes of the retinal proteome. Our data demonstrate that elevated IOP causes alterations in the retinal protein profile, in particular in high-mobility-group-protein B1 (HMGB1), calmodulin, heat-shock-protein (HSP) 70 and carbonic anhydrase II expression. The changes of the retinal proteome by dorzolamide or travoprost are different and independent of the IOP lowering effect. This fact suggests that the eye drops exert a direct IOP-independent effect on retinal metabolism. Further investigations are required to elucidate the potential neuroprotective mechanisms signaled through changes of HMGB1, calmodulin, HSP70 and carbonic anhydrase II expression in glaucoma. The data may facilitate development of eye drops that exert neuroprotection through direct pharmacological effect.

## Introduction

Glaucoma is a chronic neurodegenerative disease which is characterized by a progressive loss of retinal ganglion cells (RGCs). The elevation of the intraocular pressure (IOP) is the mayor risk factor which is associated with the progression of the chronic disease [1], [2]. Therefore current anti-glaucomatous treatment is based on reducing the IOP, thus limiting isease progression [3]. In some patients, the degeneration of RGCs continues despite of a significant reduction of the IOP, suggesting an ongoing intraretinal response that, once initiated by the elevated IOP, continues independent of it [2]. IOP may initiate a self-propagating process of RGC degeneration. As a consequence, the neuroprotection of RGC has been emphasized as an important strategy for the management of glaucoma [4]. One approach to develop a neuroprotective therapy is to identify the neuroprotective profile and the pharmacological effect on retinal ganglion cells of anti-glaucomatous drugs currently used for patients.

Among the drugs used in the clinical management of glaucoma, prostaglandin F2α analogues, such as Travoprost, have a potent IOP-reducing effect in patients with glaucoma through increasing the drainage of aqueous humour along the uveoscleral pathway [5]. Thus, they are considered to be first line therapy in glaucoma.Another first choice drug is dorzolomide which is a carbonic anhydrase inhibitor that reduces the production of aqueous humour by blocking the carbonic anhydrase in the cilliary body [6].For both drugs a neuroprotective effect has been reported [7], [8], however without elaborating on possible molecular targets within the retina. A neuroprotective drug may act to alter the retinal protein metabolism by either directly modifying the ganglion cells response to different IOP levels, or indirectly changing the glia-derived neurotrophic factors. Although the exact mechanism of the neuroprotective action remains unknown, it is thought that retinal protein metabolism is modulated in response to the IOP level.

The main purpose of the present study was to identify changes in the metabolism of the retina at proteomic level in an inherited rat glaucoma model. Then we explored whether topically applied dorzolamide and travoprost have a pharmacological effect on the proteome of rat retina distinguishable from IOP-derived changes.

## Material and Methods

### Ethical Statement and Animals

All experiments were conducted in accordance with the Association of Research in Vision and Ophthalmology (ARVO) Statement for the Use of Animals in Ophthalmic and Vision Research. The ethics committee (Bezirksregierung Münster, i.e regional government of Münster) specifically approved this study (permission-No: 50.0835.10 G9/2001). Animals were housed in a standard animal room with food and water *ad libitum* and a 12 hrs light-dark cycle.

**Figure 1:**
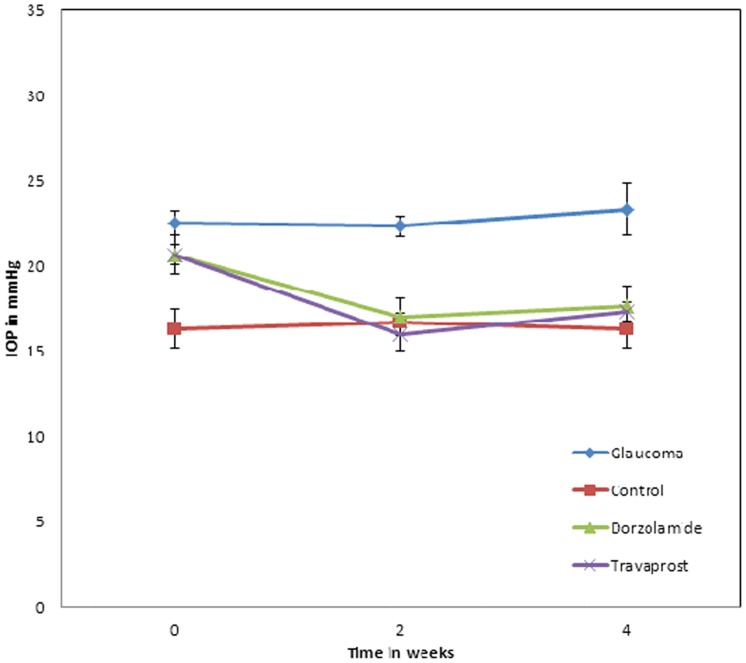
IOP readings in an inherited glaucoma rat model. The IOP was significantly elevated in all glaucoma rats compared to normal control group (red line) (* p<0.05). After treatment with dorzolamide or travoprost the IOP was reduced significantly (* p<0.05), whereas in the glaucoma group, without hypotensive treatment (blue line) the IOP remained elevated.

The animals were obtained from a colony bred in our laboratory and developing ocular hypertension [9]. The rat mutants which had developed ocular hypertension derived from the Royal College of surgeons (RCS)-strain that is characterized by photoreceptor dystrophy [9], [10], due to a mutation in the tyrosin kinase gene Mertk [11]. In order to separate the Mertk-mutation from the yet unknown mutation resulting in ocular hypertension, hypertensive rats were back-crossed with the wild type brown rats (*R. norvegicus*) to obtain descents with elevated IOP but no photoreceptor dystrophy revealed by recording electroretinograms (ERGs). Rats with normal ERGs and elevated IOP were included in this study. Rats presented with an IOP>20 mmHg over 4 weekly measurements were included into the hypertensive groups, whereas normotensive mates were considered when IOP was <18 mmHg over 4 weekly measurements.

**Figure 2:**
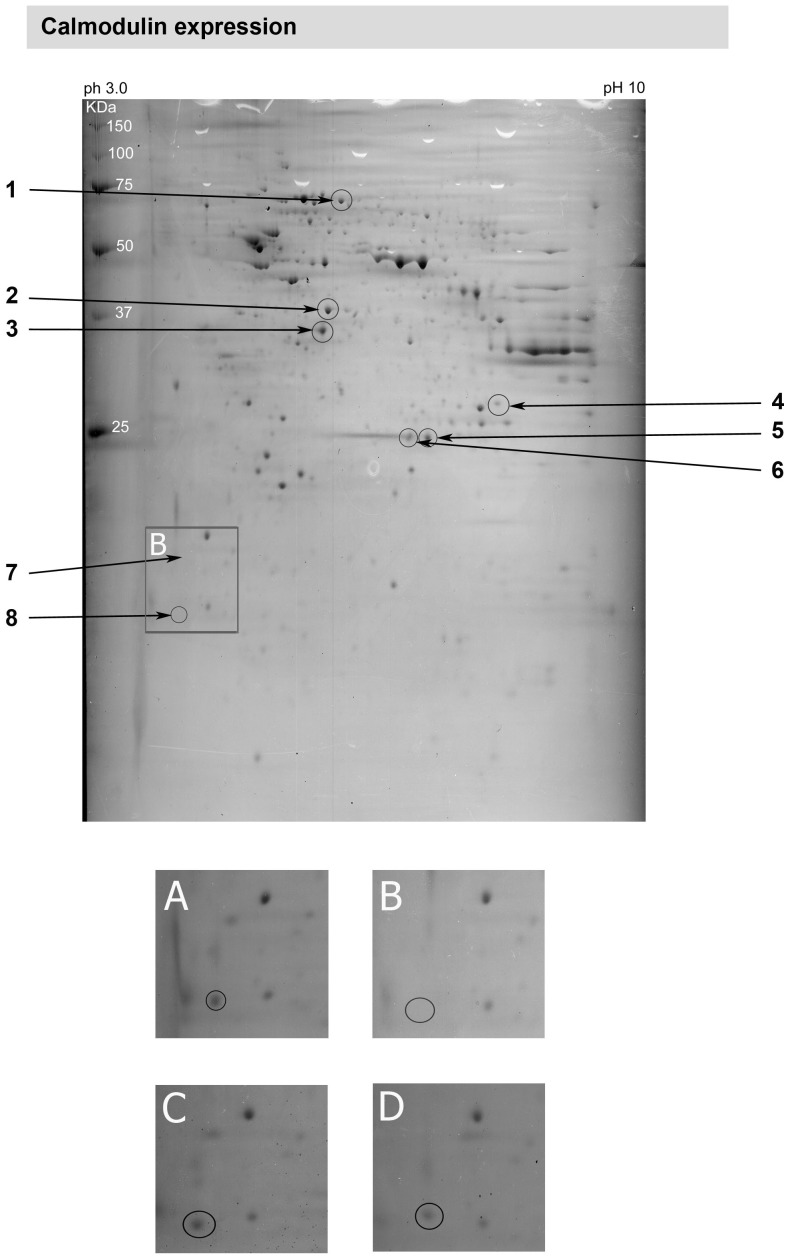
Peptide mapping of retinal explants obtained from a rat with inherited glaucoma and elevated IOP. Hypertensive retinal samples showed a marked decrease in calmodulin expression (area marked by a white box and shown in a higher magnification in B)) compared to normotensive retina A). Four weeks treatment with either travaprost C) or dorzolamide D) elevated the calmodulin expression in a similar manner, therefore normalizing its expression.

**Table 1:** Retinal proteins identified by two-dimensional gel electrophoresis and subsequent matrix-assisted laser desorption ionization mass spectrometry.

Spot #	kDa	Protein	SwissProt
**1**	70	HSP70	Q07439
**2**	37	Alpha enolase	P04764
**3**	35	Guanine nucleotide-binding protein	P54311
**4**	27	Carbonic anhydrase 2	P27139
**5**	25	High mobility group protein 1	P63159
**6**	25	High mobility group protein 1	P63159
**7**	19	Cold-inducible RNA-binding protein	P60825
**8**	17	calmodulin	P62161

The number in the left column refers to those given in Figure 2. The second column lists the molecular mass in kilodaltons. The right column lists the Swissprot number of the respective proteins.

**Figure 3:**
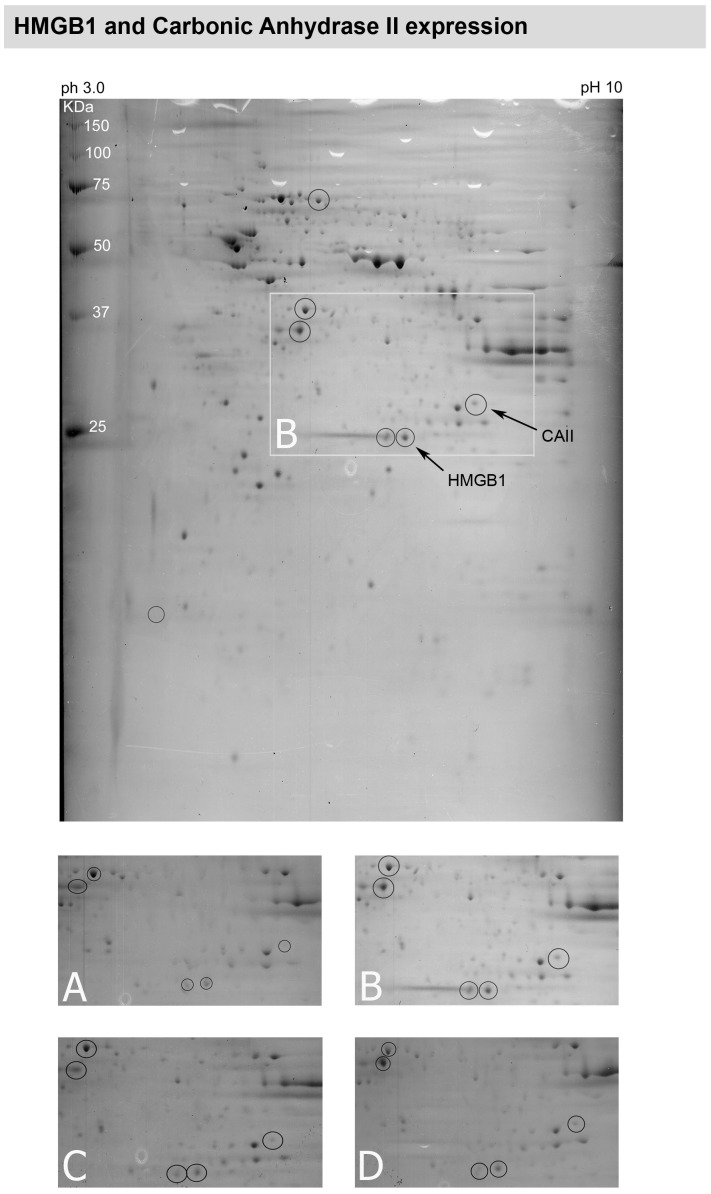
Peptide mapping of retinal explants obtained from a rat with inherited glaucoma and elevated IOP. Hypertensive retinal samples showed a marked increase in HMGB-1 expression and slightly increase in CAII expression (area marked by a white box and shown in a higher magnification in B)) compared to normotensive retina A). After 4 weeks anti-glaucomatous topical treatment with travoprost C) and dorzolamide D) HMGB-1 expression is slightly reduced in retinas treated with travaprost C) but not in retinas treated with dorzolamide D), despite of a similar reducing effect on the IOP. The expression of CA II showed nearly constant expression profile independent of treatment or not (A–D).

**Figure 4:**
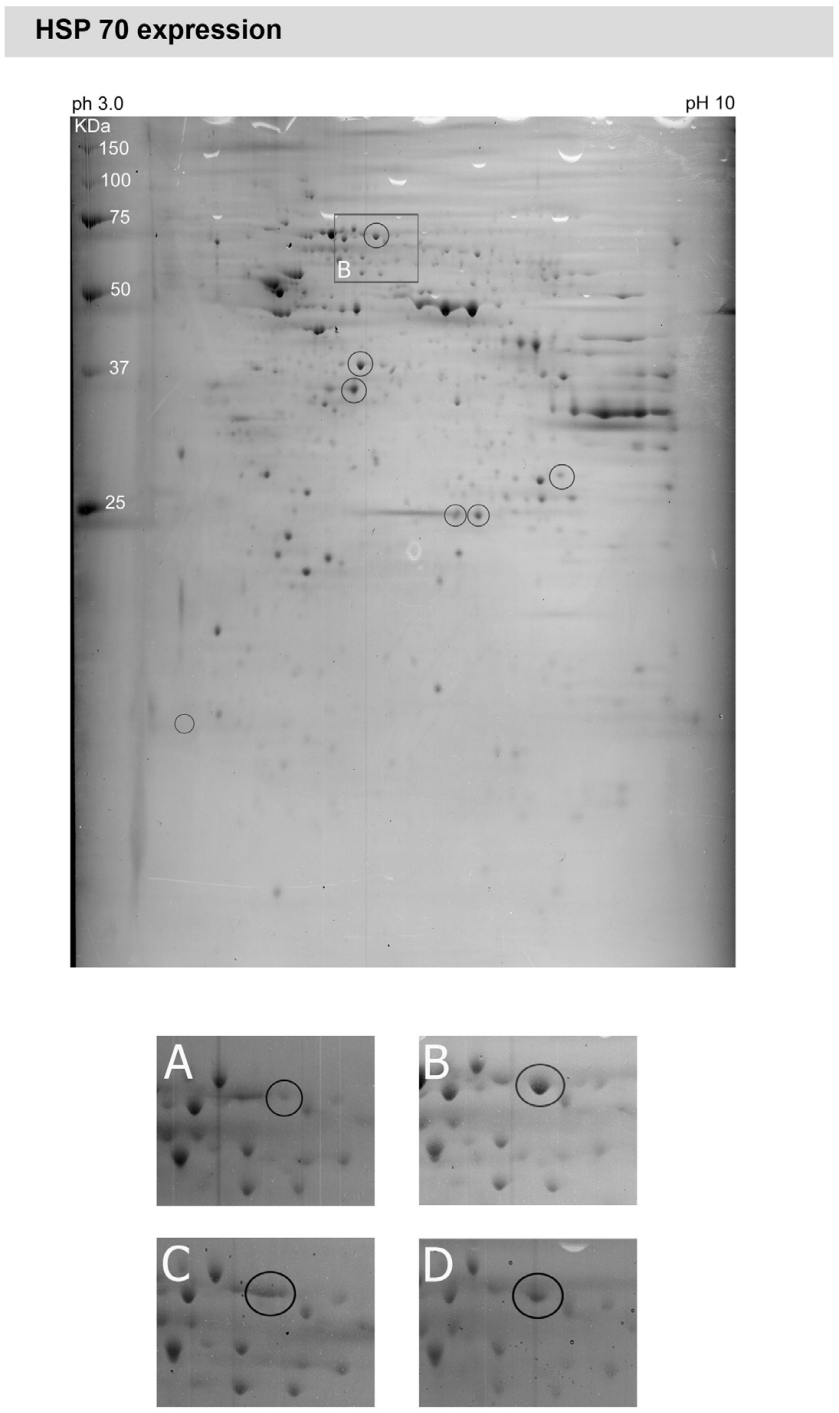
Peptide mapping of a retinal explants obtained from a rat with inherited glaucoma and elevated IOP. Hypertensive retinal samples showed a marked decrease in HSP70 expression (area marked by a white box and shown in a higher magnification in B)) compared to normotensive retina A). Four weeks treatment with either travaprost C) or dorzolamide D) slightly reduced the HSP70 expression.

### Application of the Eye Drops and Intraocular Pressure Measurement

Eye drops containing 2% dorzolamide hydrochloride (Trusopt, MSD München, Germany) or 40 µg/ml travaprost (Travatan, Alcon, Hünnenberg, Switzerland) were applied topically to the hypertensive eye daily between 8∶00 a.m. and 10∶00 a.m. over a 4-week period. The residence time of each drop was at minimum 1 minute. One group (n = 4) with high intraocular pressure remained untreated as positive control. One normotensive group (n = 4) out of the breed was used as negative control.

**Figure 5:**
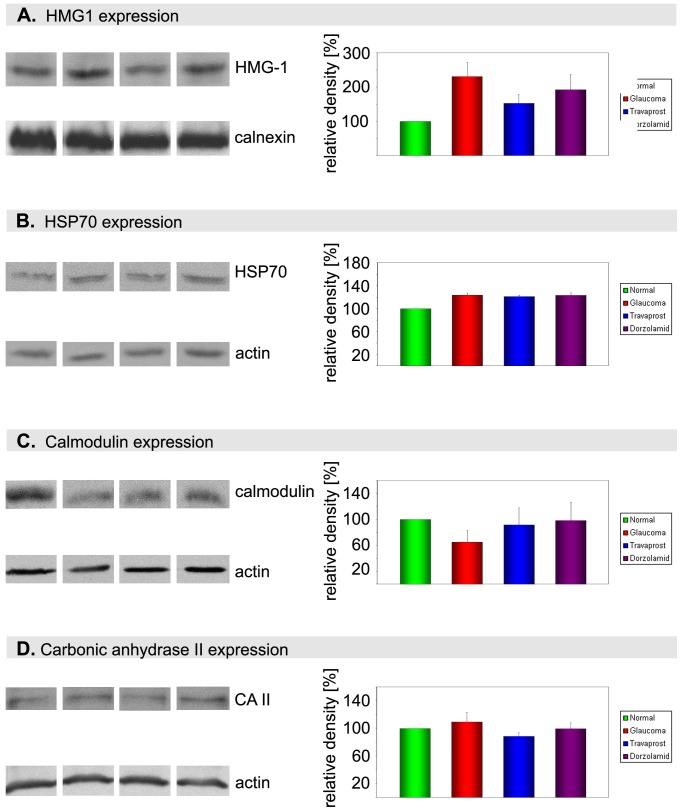
Specific Western blot analysis and the correlated graphs of the relative density of the selected protein normalized to application controls. A) Western blot showing that HMGB-1 was up-regulated in glaucomatous rats. The up-regulation was significantly reduced in retinas treated with travaprost but not significantly in retinas treated with dorzolamide (A). HSP70 showed a moderate up-regulation in glaucomatous retinas, travaprost (121% ±3) and dorzolamide treated retinas (B). Calmodulin was significantly reduced in glaucomatous retinas while this reduction was prevented in the groups treated with either drugs (C). CAII showed a clear expression in all groups without significant changes (D). Actin or Calnexin was used as a standard control in these probes. Data were presented as relative mean values ± SD. n  = 3 in rat retina. Three independent Western blots were performed. * p<0.05 and ** p<0.01.

Intraocular pressure was measured weekly while the rats were slightly anesthetized by isoflurane inhalation (Isofluran DeltaSelect, Actavis, Langenfeld, Germany). The eyes were additionally anesthetized with a drop of topical 0.5% proparacaine (Ursa-Pharm, Saarbrücken, Germany). All measurements were carried out between 9∶00 a.m. and noon using a tonometer (Tono-Pen XL, Mentor, Norwell, MA) that was calibrated before each session according to manufactureŕs instruction. On any given eye, ten tonometer readings taken directly from the display of the instrument were recorded and averaged. “Off” readings and instrument-generated averages were ignored.

**Figure 6:**
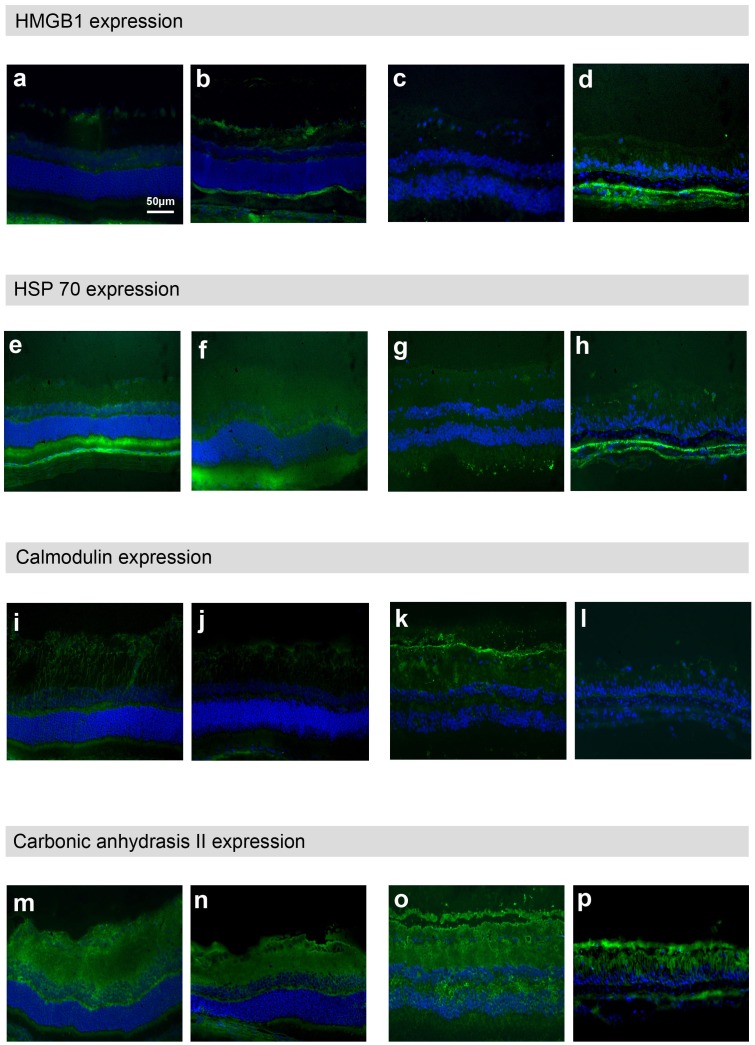
Expression of HMGB-1, HSP 70, calmodulin and carbonic anhydrase II in rat and human retinas. Immunohistochemistry of normal and glaucomatous rat retinas showing up-regulation of HMGB-1 (ab) and the down-regulation of calmodulin (i,j) expression. The immunochemistry staining of HSP 70 and carbonic anhydrase II showed no changes (e,f,m,n) in rat retina. Immunohistochemistry, showing up-regulation of HMGB-1 (c,d) and HSP 70 (g,h) and the down-regulation of calmodulin (k,l) in normal and glaucomatous human retinas.

### Histology and Immunohistochemistry

Rats were euthanized after 4 weeks of treatment and one-quarter of the retinas of the enucleated eyes were embedded in TissueTek (Tekura Finetek, Zoeterwoude, The Netherlands) for cryosection. To exclude a photoreceptor degeneration at histological level, in addition to the ERGs, tissue sections were stained with hematoxylin and eosin and examined microscopically to assess the photoreceptor layer.

Frozen sections (10 µm thick) of rat and human retina samples from normotensive eyes and glaucomatous eyes from the eye bank were used for immunochemistry staining. For the use of human retinal sections from donor eyes that were analysed anonymously according to the federal medical ethics commission for the use of human probes, the University ethics committee was asked and it approved the use of probes. Written informed consent was obtained for the tissue samples. Antibodies to stain HSP70 and calmodulin were obtained from New England Biolabs (New England Biolabs, MA) and used at a dilution of 1∶100. Antibodies to HMGB-1 and carbonic anhydrase II were obtained from Sigma and Santa Cruz, respectively, and used at a dilution of 1∶100. Cryosections were fixed in ice-cold methanol, and then cells and cryosections were incubated for 1 h in PBS containing 10% FCS to reduce nonspecific binding and overnight at 4°C with the primary antibody in PBS containing 3% FCS. After washing three times in PBS, the cells and the cryosections were incubated for 1 h in PBS supplemented with 3% FCS and either IgG conjugated to fluorochrome Cy-2 (Dianova, Hamburg, Germany) or IgG conjugated to fluorochrome TRITC (Sigma). The cryosections were mounted in mounting medium (Mowiol; Merck, Darmstadt, Germany) containing DAPI (4′, 6-diamidino-2-phenylindole; Sigma) to stain the cell nuclei. The immunofluorescence was recorded using fluorescence microscopy (Axiovert; Carl Zeiss, Oberkochen, Germany).

### 2D Gel Electrophoresis and Proteomics

The remaining three-quarters of the retinas of each enucleated eye were harvested and used for proteomic analysis using 2D-PAGE and mass spectrometry (MS) peptide mapping. 2D-PAGE was performed according to the method first described by O’Farrell [12].

In detail, the explants of each retina were boiled in 10% sodium dodecyl sulfate (SDS; Sigma, Taufkirchen, Germany) and homogenized in 2D lysis buffer (7 M urea, 2 M thiourea; Merck, Darmstadt, Germany), 4% 3-[(3-cholamidopropyl)-dimethylammonio]-1-propane sulfonate (USB, Cleveland, OH), 40 mM Trisbase (Roth, Karlsruhe, Germany), 1 mM phenylmethylsulfonyl fluoride (Sigma), and 10 mM dithiothreitol (Roche, Mannheim, Germany). The final SDS concentration was 0.25%. Soluble protein (200 µg, according to the Bradford test) together with a 2% immobilized pH gradient (IPG) buffer (pH 3–10, Amersham Biosciences, Freiburg, Germany) and 20 mM dithiothreitol were loaded on Immobiline Drystrips (pH 3–10, 18 cm; Amersham Biosciences) and rehydrated overnight. The rehydrated strips were focused on a Multiphor II (Amersham Biosciences) electrophoresis system for not, vert, similar80 kVh. Focused IPG strips were incubated twice for 15 min in equilibration solution [50 mM Tris–HCl (pH 8.8), 6 M urea, 30% glycerol, and 2% w/v SDS] and a trace of bromophenol blue (Merck), with 1% β-mercaptoethanol and 2.5% iodoacetamide (Sigma) added to the first and second equilibration steps, respectively. For the second dimension, the equilibrated IPG strips were fixed with 0.5% w/v melted agarose (Merck) on homogeneous 12.5% SDS gels (rotiphorese Gel 30, Roth). Proteins were separated by vertical SDS–PAGE (BioRad, Munich, Germany). Protein spots were initially labelled with colloidal Coomassie Brilliant Blue G250 (Merck, Darmstadt, Germany).

Spots were manually excised from the gel, tryptically digested in the gel, extracted, purified using Ziptips (microbed C18; Millipore, Bedford, MA), and subjected to MS analysis. Peptide maps were generated using a TOF-Spec-2E device (Micromass, Manchester, UK), and selected retinal peptides were sequenced using a nanoHPLC-MS/MS device (Ultimate, LC Packings, Amsterdam, The Netherlands; Esquire3000, Bruker Daltonics, Bremen, Germany). Three gel replicates were compared. National Center for Biotechnology Information (NCBI) and SWISS-PROT databases were searched using Mascot software (Matrix Science, London, UK). Additional image analyses were performed on gels stained with silver nitrate.

### Western Blotting

Freshly isolated retinas were frozen in liquid nitrogen, and then homogenized in SDS sample buffer (62.5 mM Tris-HCL, 2% w/v SDS, 10% glycerol, 50 mM DTT, and 0.01% w/v bromophenol blue). After sonicating and heating the samples, the protein concentration was determined using Bradford reagents. Fifty micrograms of protein from each sample were fractionated on 8%, 10%, or 12% SDS-PAGE (depending on the examined protein to be examined) with a protein marker (BioRad, CA). After electrophoresis, proteins were transferred to a nitrocellulose membrane. The blots were incubated in blocking solution (5% fat-free dry milk and 0.1% Tween-20 PBS) for 1 h, followed by incubation overnight at 4°C with HMGB1 (Sigma), calmodulin, HSP70 (New England Biolabs, MA) and Carbonic anhydrase II (Santa Cruz, CA) antibodies used at a dilution of 1∶1000. The membrane was then incubated with the secondary antibody conjugated with horseradish peroxidase in blocking solution for 1 h at room temperature. Antibody detection was performed with enhanced chemiluminescence (Amersham), and densitometry was performed using AlphaEase (AlphaEase FC software 4.0; Alpha Innotech). The spot density was determined for each spot after subtracting the specific background density. The spot density was correlated and normalized to the relative density of the particular application control. The untreated normotensive spot density was defined as reference mark, and the relative relationships were determined and processed.

### Statistical Analysis

All data regarding IOP recordings and relative protein densities in WBs are presented as mean ± SD values. Data were analyzed using Student’s t-test, with P values <0.05 considered as statistically significant (*) and P values <0.01 as very significant (**).

## Results

### Monitoring of Pharmacological Changes on Intraocular Pressure

The mean IOP of hereditary glaucoma rats were 23.3±1.5 mmHg. The age-matched normotensive group has an IOP of 16.3±1.2 mmHg. Under medication with dorzolamide and travaprost once-a-day the IOP was significantly decreased from 20.7±0.8 mmHg to 17.6±1.2 mmHg (P<0.05) and from 20.7±0.7 mmHg to 17.3±0.6 mmHg (P<0.01), respectively. These recorded readings remained constant over 4 weeks of antihypertensive treatment. IOP recordings are illustrated in Figure 1.

### Topical Medication Influences Proteomic Profile

MS-assisted peptide analysis of retinas from normotensive rats, glaucomatous rats and rats which were treated with dorzolamide and travaprost respectively revealed that some proteins were differentially expressed within the retina. Figure 2A shows the scattered proteins in a two-dimensional gel. Table 1 lists the spot number, name, SwissProt number and molecular weight of each assessed protein. When considering the region enframed in figure 2, the spot encircled is well expressed in the normal retina (Fig. 2A) and down-regulated in the hypertensive retina (Fig. 2B). Treatment with either travaprost (Fig. 2C) or dorzolamide (Fig. 2D) elevated the calmodulin expression in a similar manner, therefore normalizing its expression. The proteins which were differentially expressed within the different retinas are marked. The high mobility group protein box 1 (HMGB-1) is a protein which is known to be involved in coping with different stresses [13] and was up-regulated in glaucomatous retina (Fig. 3B). The effect of elevated IOP was slightly reduced in retinas treated with travaprost (Fig. 3C) but not in retinas treated with dorzolamide (Fig. 3D), despite of a similar reducing effect on the IOP (Fig. 1). The expression of carbonic anhydrase 2 (CA II) (Fig. 3) which is a specific Müller cell marker showed nearly constant expression profile independent of treatment or not (Fig. 3A–D). Another protein which is up-regulated by elevated IOP was HSP70 (Fig. 4). HSP70 was strongly elevated in the hypertensive retinas (Fig. 4B) to be slightly reduced after lowing the IOP in either dorzolamide (Fig. 4C) or travaprost (Fig. 4D) treated eyes. The induction HSP70 expression is known to be neuroprotective in retinal ganglion cells [14].

### Confirmation and Quantification with Western Blotting

Additional Western blot analysis of HSP70, calmodulin, HMGB-1 and carbonic anhydrase II was performed to confirm the results of the proteomic analysis. First, HMGB-1 was examined in controls, glaucomatous retina and after treatment with dorzolamide and travaprost, respectively. It appeared that HMGB-1 is expressed in untreated controls and strongly up-regulated in glaucomatous retinas (207% ±59). The HMGB-1 upregulation was significantly reduced in retinas treated with travaprost (139% ±46) but not significantly in retinas treated with dorzolamide (164% ±61) (Fig. 5A). These results support the findings of the 2D gel electrophoresis. The western blot analysis of HSP70 showed a moderate up-regulation in glaucomatous retinas (123% ±3), travaprost (121% ±3) and dorzolamide treated retinas (123% ±4) (Fig. 5B). However, the changes were not significant calmodulin was significantly reduced in glaucomatous retinas (65% ±17) while this reduction was prevented in the groups treated with either drugs (Fig. 5C). CAII showed a clear expression in all groups without significant changes. These data confirm the proteomic data at western blot levels.

### Localization of HSP70, Calmodulin, HMGB-1 and Carbonic Anhydrase II in Rat and Human Retina

To determine cellular localization of the identified proteins (HSP70, calmodulin, HMGB-1 and carbonic anhydrase II) within retinal sections and to confirm their expression at histological level immunohistochemistry of rat and human retina sections was performed.

All proteins occur within human and rat retina. HMGB1 is slightly expressed in normal rat (Fig. 6A) and normal human retina (Fig. 6C). In glaucomatous eyes HMGB1 staining was stronger and predominantly in the ganglion cell layer and the external limiting membrane of rat (Fig. 6B) and glaucomatous human retina (Fig. 6D). HSP 70 is expressed in normal rat (Fig. 6E) and human (Fig. 6G) retina as well in rat (Fig. 6F) and human (Fig. 6H) retinas suffering from glaucoma. Calmodulin is predominantly stained in the inner plexiform layer of both species (Fig. 6I, K). In both species calmodulin staining was weaker in glaucomatous retinas (Fig. 6J, L). Carbonic anhydrase II staining was similar in all retinas without showing significant changes. (Fig.6M–P). The immunhistochemical data confirmed the proteomic changes and helped to unravel the localization of each protein within retinal sections of normal and glaucomatous tissue. In addition, these data showed that rat and human retinas show similar protein profiles when exposed to elevated IOP.

## Discussion

The principal findings of this study are that (1) elevated IOP modulates the pattern of protein expression in the inherited glaucoma rat model; (2) treatment with dorzolamide or travaprost is effective at lowering IOP; (3) dorzolamide and travaprost exert additional pharmacological effects on the retinal proteome which are independent of IOP changes. These novel data indicate that topically applied eye drops are able to change the retinal metabolism. The retinal proteins assessed may provide tools for studying the mechanisms of cell death in glaucoma and for preventing disease-associated neuropathies. Further studies are needed to develop topical antiglaucomatous eye drops with neuroprotective properties.

Although glaucoma is a leading cause of blindness worldwide, affecting about 2% of individuals of European descent and up to 10% of individuals of sub-Saharan African descent over 50 years of age, the exact molecular mechanisms of the IOP-induced optic neuropathy remain unrevealed [15]. Elevated IOP is a major risk factor for glaucoma. As a consequence, current treatments are primarily focused on reducing the IOP [3]. However, RGC cell loss is usually, but not always, associated with elevated IOP [16]. Immunmodulatory and vascular factors has been identified to play a crucial role in glaucoma development [17]. Therefore, the neuroprotection of RGC has been emphasized as an important strategy in managing glaucoma [4], [18].

The proteomic analysis of ocular hypertensive retina from the inherited glaucoma rat revealed several proteins which were differentially expressed. Four major proteins specific to ocular hypertensive retina were identified by mass spectrometry: HMGB1, a non-histone nuclear protein with dual function; HSP70, a molecular chaperone and stress protein; calmodulin, a Ca^++^-binding protein; and carbonic andyrase II, a zinc metalloenzym which catalyze the reversible hydration of CO2 to form HCO3^−^ and protons.

One of the differently expressed proteins, HMGB1, is known to be ubiquitously expressed in mammalian cells, mainly in the cell nucleus [19]. Several lines of evidence suggested HMGB1 as a pathophysiologically active mediator of lung and liver disease as well as sepsis [19], [20]. On the other hand HMGB1 exhibits beneficial effects in a model of myocardial infarction [21].

In neuronal cells it has been reported that extracellular HMGB1 aggravates the tissue damage in ischemic brain infarction models [22], [23]. In addition to these studies, it has been shown that HMGB1 inhibits glial glutamate transport and increase extracellular glutamate concentration that may cause neuronal excitotoxicity [24]. The role of HMGB1 in glaucoma is still obscure.

The results of our proteomic and Western blot analysis revealed that HMGB1 expression is up-regulated in the retina exposed to elevated IOP in the inherited glaucoma rat model. In addition, the result of our immunohistological staining displayed that HMGB1 was stained in glaucomatous rat and human retina. These results suggest that HMGB1 may play a role in degenerative events initiated by IOP-elevation, both within the retinal cells and the extracellular space.

HSP 70 was another protein up-regulated in retina of glaucomatous eyes. HSPs are molecular chaperones involved in several cellular processes including stress response [25]. Members of the HSP70 family have been suggested to play role as native defense mechanism in response to cellular stress in glaucoma *in vitro* [26]. Furthermore, the induction of a protein of HSP 70 family by heat stress, systemic zinc application or geranylacetone application, provided neuroprotection in an experimental glaucoma rat model [14], [27]. The molecular function of HSP 70 has been suggested to bind to Apaf1 and prevent recruitment of caspases to the apoptosome complex. Hsp70 suppresses apoptosis by blocking the assembly of a functional apoptosome [28]. Antibodies against HSP 70 and other HSPs were detected in glaucomatous eyes [29], [30] indicating that the protein induces an autoimmune response.

Calmodulin (CaM) was down-regulated in the retina of hypertensive eyes in the inherited glaucoma rat model. The ubiquitous calcium-sensing protein calmodulin is involved in several cell signaling pathways [31], [32]. Activated Ca^2+^-calmodulin complex binds to Ca^2+^-Calmodulin-dependent protein kinases (CaMKK) and activates pro survival pathways [33], [34]. There are several lines of evidence that neurotrophic factors such as BDNF or GDNF provide their neuroprotective capacity through increased intracellular Ca^2+^ or direct regulation of PI-3-kinase activity by CaM [31], [35]. The deprivation of neurotrophic factors is thought to be one of the most important pathophysiological mechanisms of glaucoma [36], [37]. On the other hand, recent studies showed that some isoforms of CaMKK are able to regulate the BDNF expression in neuronal cells [38], [39].

The results of our proteomic and Western blot analysis revealed that calmodulin expression is down-regulated by elevated IOP in the inherited glaucoma rat model. In addition, the result of our immunhistological staining displayed that calmodulin was predominantly apparent in the inner plexiform layer which is the major layer of synaptic transmission from interneurons to RGCs. In human and rat retina suffering from glaucoma calmodulin staining was weaker. These results showed that down-regulated calmodulin expression is associated with the glaucomatous damage and may be caused by either the neurotrophic factor deprivation, or the reduction of synaptic density due to cell decay.

In addition to the IOP induced alterations of the retinal proteome in the inherited glaucoma rat model, we found a drug-specific and IOP-independent regulation of HMGB1 and calmodulin. The HSP70 expression is not affected by the applied antihypertensive drugs despite of the IOP-lowering effect.

For the drugs used such as dorzolamide and travaprost neuroprotective capacity has been suggested in several studies. Prostaglandin F_2α_ analogues may exert their neuroprotective effects via the prostaglandin F receptor [40]. Other studies showed that the neuroprotective effect might be through the suppression of cyclooxygenase (COX-2) activity or other ways which may be not related to FP receptor stimulation [8], [41]. Carbonic anhydrase inhibitors are suggested to augment the retrobulbar blood flow in glaucoma patients and act neuroprotective in this way [42]. Kniep and co-workers showed a direct anti-apoptotic effect of dorzolamide against advanced glycation end products induced apoptosis [43]. For both drugs the exact mechanism of the neuroprotective effect remains unclear. Our data show that dorzolamide and travoprost induce retina metabolic changes that are independent of IOP.

In conclusion, our study demonstrates that elevated IOP causes alterations in the retinal proteome in particular in HMGB1, calmodulin, HSP 70 and carbonic anhydrase II expression. The changes of the retinal proteome by dorzolamide or travoprost are different and independent of the IOP lowering effect. This fact suggests that the eye drops exert a direct IOP-independent effect on retinal metabolism. Further investigations are required to elucidate the potential neuroprotective mechanisms of HMGB1, calmodulin, HSP70 and carbonic anhydrase II in glaucoma and develop eye drops that exert neuroprotection through direct pharmacological effect.
